# Adaptation of the WHO group interpersonal therapy for people living with HIV/AIDS in Northwest Ethiopia: A qualitative study

**DOI:** 10.1371/journal.pone.0238321

**Published:** 2020-08-27

**Authors:** Biksegn Asrat, Crick Lund, Fentie Ambaw, Marguerite Schneider

**Affiliations:** 1 Alan J Flisher Centre for Public Mental Health, Department of Psychiatry and Mental Health, University of Cape Town, Cape Town, South Africa; 2 Centre for Global Mental Health, King’s Global Health Institute, Department of Health Services and Population Research, Institute of Psychiatry, Psychology and Neuroscience, King’s College London, London, United Kingdom; 3 School of Public Health, College of Medicine and Health Sciences, Bahir Dar University, Bahir Dar, Ethiopia; 4 Department of Psychiatry, College of Health Sciences, Addis Ababa University, Addis Ababa, Ethiopia; Fordham University, UNITED STATES

## Abstract

**Background:**

Psychological treatments improve depressive symptoms in people living with HIV/AIDS (PLWHA). Adaptation of treatments should be based on explanatory models of depression and other elements within the given context.

**Aim:**

This study aimed to examine explanatory models of depression and acceptable approaches for implementation of group IPT in Northwest Ethiopia.

**Methods:**

Qualitative data were collected from April to May 2019 from case managers, adherence supporters and service users using focus group discussion and analysed thematically.

**Results:**

PLWHA attributed depression to psychosocial problems, spiritual factors and biological factors. Depression had several impacts at individual and family level. Group-based interpersonal therapy (IPT) was acceptable if provided by trained peer counselors.

**Conclusion:**

The current study findings informed how to conduct feasibility and acceptability trials of group IPT in the HIV population in Ethiopia.

## Introduction

A number of psychological treatments show benefits for improving depressive symptoms among different groups of people [1, 2]. However, when they are implemented in different settings, they may not appear as effective as they were during their development [2–5]. This indicates the need for dynamic adaptation and contextualization of psychological treatments when they are replicated in different settings [2, 4]. The use of Western treatment models in diversified settings using an etic approach can invalidate psychological treatments [6]. Chowdhary and colleagues support this argument in a systematic review showing that trials following proper adaptation and contextualization of psychological treatments were more effective than trials that used non-adapted treatments [7]. The adaptation of evidence-based psychological treatments requires appropriate understanding of local explanatory models and contextualization of preferred treatments into the existing health care systems [6–8]. Although several studies have shown the effectiveness of psychological treatments for depression in LMICs, the attention given to appropriate adaptation remains limited [8].

Studies have shown that group interpersonal therapy (IPT) has been effective for treatment of depression in LMICs [9–11]. However, there is a challenge to integrating group IPT into the routine health care systems because of a lack of systematic adaptation and contextualization of the therapy [8]. There are several issues that should be considered while adapting group IPT for treatment of depression in LMICs [12]. Furthermore, engaging stakeholders in the process of contextualization of the group IPT is fundamental to enhance its acceptability and effectiveness [6, 8, 12]. In relation to people living with HIV/AIDS (PLWHA), there are several positive results on the effectiveness of group IPT for treatment of depression in LMICs [11–14]. However, systematic adaptation of group IPT specifically for PLWHA has received minimal attention to expand the intervention to larger populations.

The Kleinman’s explanatory model of illness remains a useful approach to integrate cultural beliefs within clinical processes by exploring views of therapists and patients on the nature, name, cause, course and desired treatment for a specific episode of illness [15–17]. This model views cultural and psychosocial variables as important determinants of health [18], and it helps to understand and integrate stakeholders views within health care systems [16].

Symptoms of depression and HIV/AIDS are intertwined with each other [6, 19]. For instance, several narratives indicate that PLWHA consider depressive symptoms as part of HIV/AIDS [6, 14, 19] and they believe that depression is caused by HIV/AIDS [14, 19]. Moreover, many others attribute depression to spiritual possession and witchcraft [6, 14]. Similarly, PLWHA may have different beliefs on the impacts of depression and treatment needs including on the preferred approach of interventions and delivering agents for the intervention.

Evidence shows that depression is caused by biological, psychological or/and social vulnerabilities [20–22]. The biological process of HIV such as “cytokine-induced sickness behavior” and immune activation markers can cause onset of depressive features in PLWHA [23–25]. Furthermore, the life threatening nature of HIV/AIDS has a psychological impact on PLWHA leading to low self-esteem and self-stigma that can precipitate development of depression [21, 26]. The social aspect of PLWHA is complicated with difficult life circumstances such as public stigma and poverty [27]. As a result, several studies indicated that depression can be caused by 1) interpersonal problems such as separation, conflict, stigma and loneliness [28, 29], and 2) life changes in relation to the HIV/AIDS such as sickness, unemployment and poverty [30–32].

The origin of most triggering factors of depression are understood within IPT as mainly social problems that are then the focus of treatment (problem areas) of IPT [12, 14]. IPT hypothesizes that depression can be treated when social support is enhanced and interpersonal stress is resolved [33]. Other scholars suggest including spiritual and cultural components into treatment models of depression [34, 35] and mental health care services [36] since they are important aspects for treatment of many people, especially at the end of life [35]. However, further studies are needed on how to address the spiritual elements within IPT specifically for PLWHA in LMICs.

Studies from sub-Sahara Africa have shown that depressed PLWHA prefer group-based psychological treatments [11, 14, 29, 37, 38] as these provide supportive environments and help participants to learn new social skills [11, 29]. Furthermore, providing or receiving social support during group sessions gives one a sense of belonging and meaning in life [11, 14]. Participants report gaining a feeling of acceptance, sense of empowerment and confidence when they are in a group [29]. This implies that group-based interpersonal therapy can be a potentially acceptable option for treatment of depression for PLWHA in Ethiopia.

In Ethiopia, IPT has never been adapted for PLWHA. Adaptation is an essential initial step for testing the feasibility and acceptability for this target population in Ethiopia. This paper focuses on examining the explanatory models of depression and acceptable contexts for implementation of group IPT, such as appropriateness, preferred treatment providers and perceived barriers for the delivery of the group IPT in the HIV care setting. This information is essential for the overall adaptation process.

## Materials and methods

### The process of adapting group IPT

The World Health Organization (WHO) developed the group IPT manual in 2016 [12] to provide guidance on the implementation of group IPT for treatment of depression. The WHO recommends adaptation of the manual for different settings and groups of people. We received approval from the WHO to translate and publish the manual in the Amharic language (Agreement letter TR/18/041). The WHO group IPT manual was adapted over a 5-month period from April to August 2019.

Initially, the authors of this paper reviewed existing literature to identify effective psychological treatments for depression for PLWHA in LMICs [39]. From this systematic review we found that interpersonal therapy is effective for treatment of depression especially for people with psychosocial problems. We also learned that there is a culture of conducting group HIV education in our study setting in Northwest Ethiopia. The adaptation process of the group IPT was guided by the ecological validity and culture sensitivity model [40]. Furthermore, we used Kleinman’s explanatory model of illness to understand broader views of PLWHA on depression [15].

### Setting

This study was conducted in Felege-Hiwot Referral Hospital (FHRH) which is located in Bahir Dar, Amhara regional state in Northwest Ethiopia. Bahir Dar is the capital city of the regional state that has a population of more than 750,991 residents. According to the Central Statistical Agency of Ethiopia, 96.8% of the population speaks Amharic as their first language and 93.2% are from Amhara ethnic group. Of the total population in Bahir Dar, 79.7% are Ethiopian Orthodox Christians, 18.47% Muslims, and 1.62% Protestants [41]. FHRH is one of the busiest hospitals in Ethiopia and serves a population of more than 7 million people with 430 beds [42] and 422 health professionals [43]. Of the total health professionals, 107 were medical doctors, 174 were nurses and 7 were mental health professionals [42]. The hospital records show that 6,251 PLWHA have attended the ART clinic for their treatment over the last six months. People living with HIV/AIDS play a significant role in the HIV/AIDS care and treatment in the clinic as paid case managers and adherence supporters. Case managers and adherence supporters are HIV positive and they are considered as supportive staffs at the ART clinic. Case managers provide mainly HIV counselling and adherence supporters are responsible for client tracing and home visits.

### Participants’ recruitment

Eligible participants for focus group discussions were adults who were 18 years old or older and currently receiving ART treatment at the clinic, and included case managers, adherence supporters and users of the services. We aimed to conduct separate focus group discussions in each group. Study participants were assigned into three focus groups: a focus group of case managers (6 participants), adherence supporters (5 participants) and service users (7 participants). For case managers’ and adherence supporters’ groups, we selected participants who were working on the day of data collection. In addition, for service users’ focus group, we selected seven eligible participants randomly who were 18 years and above, and not severely ill from the list of participants waiting for treatment on the day of the data collection. Focus group discussions were conducted in a private space in the ART clinic and all of them were led by the first author (BA). All the focus group discussions were conducted in the local language (Amharic) and lasted from one and a half to two hours.

### Data collection

We used focus group discussions to collect information from participants. Focus group discussions were facilitated using an open-ended interview guide. The interview guide was informed by the framework of Kleinman’s explanatory model of illness [16]. After initial review, the authors decided to expand the interview guide to include information related to acceptable contexts for implementation of group IPT in the HIV care setting, such as appropriateness of the group IPT and perceived barriers. The interview guide was further revised and translated into Amharic (S1 and S2 Files).

We used a case vignette of major depressive disorder to initiate conversation during the focus group discussion (S3 and S4 Files). The case vignette was selected from one of the case studies presented by Sai and Furnham [44] that was used for detection of depression. The selected case was adapted to the local context by BA and FA and further reviewed by MS. After that, the case was translated into the local language by BA and it was reviewed by FA for its consistency with the initial case.

The focus group facilitator read the case study (*Mrs*. *Selam*) of a major depressive disorder twice (see the case study at the annex) and a printed case description was given to each participant to read once before starting the discussion (17 of the 18 participants were able to read and write). Afterwards, participants were asked to give their general opinion about the case of *Mrs*. *Selam*. Participants were probed to name the problem that *Mrs*. *Selam* may have, what causes the condition, what other symptoms they know about the condition, its severity, where people with Mrs *Selam’s* condition look for treatment and what impacts it could have specifically for PLWHA.

Furthermore, participants were asked to give their opinion on what psychological treatments are and whether they know of any kind of group counselling. Participants were asked for their opinions on the acceptability of group counselling specifically for PLWHA, who could facilitate the group counselling sessions, how the group should be structured in terms of group composition, group size, session frequency and meeting days, how it would be conducted in the ART clinic without affecting the regular service and what barriers could affect the implementation of group counselling in the ART setting in general.

All focus group discussions were audio-recorded, and the facilitator took notes as needed, for example, to document emotional changes among the focus group participants during the discussion. At the end of the discussion, the facilitator summarized the discussion points and acknowledged all participants for contributing their opinion in the focus group discussion.

### Data management and analysis

Audio-recorded data were transcribed verbatim by the first author (BA) and a research assistant (HT) in the local language (Amharic). Afterwards, the transcripts were translated into English and double checked for accuracy. Transcripts were imported into NVivo 12 and data was thematized using a framework analysis. Initially, nodes were created based on the framework of Kleinman’s explanatory model of illness [16]. The drafted nodes informed by the framework were naming of the condition, perceived causes, perceived symptoms, perceived severity, impacts of the condition and treatment preferences. We also added acceptability, preferred providers and acceptable context nodes for implementation of group IPT in the ART setting. Sub-nodes emerged from most of the initial nodes. For example, sub-nodes that emerged from perceived cause of depression were biological, psychological, social and spiritual causes. In addition, perceived symptoms were sub-coded as affective, behavioral and somatic symptoms. Word count and word cloud were generated to identify the most important themes. Data coding and theme identification was done by two authors (BA and MS).

### Ethical approval

A proposal of this qualitative study was approved by both the University of Cape Town’s Human Research Ethics Committee (HREC reference No. 653/2018) and Bahir Dar University College of Medicine and Health Sciences’ Ethics Committee (reference No. 007/2018). A permission letter was obtained from the Amhara Public Health Institute. All study participants provided written informed consent. One of the participants was illiterate and she signed by fingerprint after a witness read the written informed consent for her. Refreshments were served during the discussion. Each study participant received compensation to defray transport costs.

## Results

### Demographic characteristics of study participants

A total of 18 adult PLWHA (included from case managers, adherence supporters and service users) participated in the focus group discussions. The average age of the focus group participants was 38, 34 and 34 years for case managers, adherence supporters and service users respectively. In terms of gender, one of the six case managers, two of the five adherence supporters and three of the seven service users were male. The average duration of ART treatment was 7.8, 5.5, 7.0 years for case managers, adherence supporters and service users respectively. All case managers and adherence supporters had secondary level education (see Table 1).

**Table 1:** Sociodemographic characteristics of focus group participants, N = 18.

No	Gender	Age in years	Educational status	Duration of ART treatment in years	Role at the ART clinic
CM1	Female	33	10^th^ grade	9	Case manager
CM2	Male	40	10+2	6	Case manager
CM3	Female	40	10^th^ grade	8	Case manager
CM4	Female	35	11^th^ grade	12	Case manager
CM5	Female	36	10^th^ grade	3	Case manager
CM6	Female	45	10+3	9	Case manager
AS1	Female	21	10+2	2	Adherence supporter
AS2	Female	35	10^th^ grade	10	Adherence supporter
AS3	Male	58	11^th^ grade	8	Adherence supporter
AS4	Male	Refused to disclose	Refused to disclose	Refused to disclose	Adherence supporter
AS5	Female	22	10^th^ grade	2	Adherence supporter
SU1	Female	42	Illiterate	Cannot remember	Service user
SU2	Male	22	11^th^ grade	4	Service user
SU3	Female	19	10+2	2	Service user
SU4	Male	48	10+1	5	Service user
SU5	Female	18	11^th^ grade	Since birth	Service user
SU6	Female	38	9^th^ grade	2	Service user
SU7	Male	48	9^th^ grade	11	Service user

CM–case manager, AS–Adherence supporter, SU–Service user.

### Explanatory model of depression

#### Naming the problem

Participants debated on naming the condition in the presented case study. Many of them explained that the condition presented in the case vignette is called ‘Dibirt’ [depression] and ‘Ye-Dibirt chigir’ [depression problem].

*AS3*: *“We cannot bring another name for this condition*, *this is ‘Dibirt’ [depression]*. *If someone shows symptoms such as timidity most of the time*, *lost interest in everything and hopelessness in every simple problem so that s/he has depression (Dibirt)”*. A 58 years old male participant who completed high school education.

*CM4*: *“I think it can be named as ‘Ye-Dibirt chigir’ [depression problem]*. *Because the symptoms could be linked with different problems including HIV*, *imprisonment and any more problems*. *… Sadness*, *hopelessness and suicidal ideation appears when multiple problems are occurring on person*”

A few participants reported that mental health professionals call depression differently, that is ‘Medebet’ [similar meaning for depression].

*AS1*: *“In general*, *I call it ‘Dibirt’ [depression] but there are people (mental health professionals) who call it ‘Medebet’*”.

#### Perceived causes of depression

The perceived causes of depression reported by study participants included biological, psychological, social and spiritual elements (see Table 2).

**Table 2:** Perceived causes of depression among PLWHA using biopsychosocial-spiritual model.

Biological	Psychological	Social	Spiritual
• Generalized physical illness • HIV/AIDS • ART medication • Change in body image	• Thinking too much • Excessive worry • Psychological problems • Sexual abuse • Self-stigma • Witnessing traumatic incident	• Problematic relationship/unfaithfulness • Divorce/separation • Poverty • Family pressure/death of loved one • Conflict • Stigma by health professionals and community members • Failure to succeed • Loneliness • Self-stigma	• Evil attack/ spirit possession • Failure to fulfil rituals • Standing on “Atela” [local beer residue], walking on ash

Problematic relationships were the most reported causes of depression as described in the quotes below.

*CM6*: *“Married people could be HIV discordant*. *One would be HIV positive and the other may be negative*. *When they get divorced*, *the one who is HIV positive will have a difficult life*. *Then s/he becomes very sad*, *desperate*, *guilty saying ‘I am divorced because I am HIV positive’*. *They regret a lot*, *thinking how beautiful their previous life was and finally they could be sick*.”

*AS3*: *“This could happen because of problematic love*. *For example*, *she could be hurt if her husband is in love with another woman somewhere*. *This would have two problems for Selam*. *One thing*, *his unfaithfulness stresses a lot; and secondly*, *she would have economic problems*. *All these together would put big pressure on her*. *If she is economically dependent on him*, *she will not get a chance to discuss such difficult issues with him*. *Because she may afraid that he could leave her alone*.”

Public stigma was commonly reported stressor that is believed to cause depression.

*AS2*: “… *when our kids play with other kids outside home*, *they have been insulted with bad words such as* “*Ye-AIDSam Lije*” *[son of AIDS]*.

Another participant reflected the same idea in relation to public stigma.

*CM4*: *“… we have unique names in our village which was labeled in relation to our HIV status*”.

Many of the study participants highlighted that they faced the worst stigma from health professionals, family and from neighbors. The following two quotes show how much health professionals stigmatize PLWHA when providing medical care to them.

*CM1*: *“I faced a similar problem when I gave birth in a hospital*. *…*. *I was on the couch to give birth*. *When midwives recognized that I am HIV positive*, *they put off their gloves and they left me alone on the delivery couch [tear shed]*. *…*.. *Someone requested me to leave the couch and they moved me to the corridor*. *…… In the meantime*, *labor was pushing and placenta was descending*. *…*. *I gave birth at the corridor assisted by one student*.”

*CM3*: *“Similar to others*, *I was stigmatized by my family*. *…I gave birth to a baby girl after I was diagnosed with the HIV/AIDS*. *I was not breast feeding her and later she was not growing well*. *She did not gain weight*, *instead she started losing weight*. *Afterwards*, *my family members were blaming me saying ‘you infected your baby girl with HIV/AIDS’*. *…*.. *I was stigmatized a lot*. *I felt lonely*, *sad*, *disturbed and worried a lot until she diagnosed negative from HIV*.”

Many focus group participants associated mental health problems, including depression with spiritual possession and evil attack.

*AS1*: “*People associate the cause of the problem with* ‘*Atela lay mekom*’ *[evil attack while standing on local beer residue]*, ‘*Be’amed lay mehed*’ *[walking on ash] and they also link it with ‘Ye’aganint likift’ [evil attack]*.

*SU1*: *“Mrs*. *Selam probably received a ritual tradition from her ancestors*. *She could be attacked by a spirit When she failed to fulfil the ritual*.”

Adherence supporters and case managers attributed depression to psychological problems.

*AS1*: *“I believe that we are creating our problems in our mind*. *…*.. *this happens because of our incorrect thinking about ourselves and others*.”

In relation to biological factors, focus group participants attributed depression to HIV/AIDS and medications. They believe that HIV/AIDS made them sensitive to simple stressors and taking HIV medications for ever makes them become hopeless to enjoy life.

*CM4*: *“There is HIV in our blood which is a big problem*. *That is why we cannot handle simple problems*. *HIV/AIDS has an association with our weak psychology*. *…*.. *I think HIV and depression have a relationship*.”

*AS3*: *“Taking ART medications is stressful and makes you feel sad*. *……one of the causes for our stress is the disease itself”*.

*SU4*: *“Hopelessness comes from taking the ART medications for life long*. *We are expected to follow a scheduled medication regimen strictly*. *That is very stressful*.”

#### Perceived symptoms of depression

The participants predominantly described affective and behavioral symptoms. The affective symptoms predominantly reported were hopelessness, depressed mood, loss of interest, being sad, helplessness, and irritability.

*CM1*: *“…*.. *I was crying day and night holding my baby on my arm*. *I was extremely sad and worried*, *no food and drink in my mouth*. *There were many people around me such as my husband and family were with me*, *everything was available*, *but I was feeling hopelessness and helplessness*.”

Frequently reported behavioral symptoms were loneliness, not eating meals, difficulty sleeping, timidity, being violent, restlessness, sitting in one place for long time, talking slowly, suicidal attempt, looking downward and poor self-hygiene.

*CM2*: *“I have seen Selam’s problem on me*. *I was living and sleeping alone when I was sick*. *I was not sure I can woke up on the next day*. *I was saying to myself that I could die while I am sleeping so that I should sleep properly*, *stretching my legs and hands forward*. *At least my dead body should be found in a proper position*. *… I had never eat*, *I had no interest to eat at all*. *I felt I was the only person straggling with this much problems in this world*. *I used to write letters mentioning my father’s and mother’s name*, *my uncles’ names*, *their address details*, *and I put the letter in my pocket when I went to bed for sleep*. *At least they should know that I died because of natural cause*.”

Another case manager said, *CM4*: *I know when clients are depressed*. *They become angry*, *irritable*, *they have no patience*, *they fight and try to strangulate health professionals with no reason*. *This is not their fault because they are sick*. *During interviews*, *they do not tell us that they are sick but they lose weight from time to time*, *they are worrying and their behaviour becomes unstable*.

Somatic symptoms were also reported by study participants, such as body pain, headache, hearing a noise in the head as if insects are moving in their head (chichichi) and bell ringing in the head. Cognitive symptoms of forgetfulness and loss of attention were frequently reported by participants.

*CM6*: *“When I was depressed*, *I used to boil water to pour it on me*. *I was trying to end my life several times*. *I used to have chronic headache*. *… It was a difficult time*, *I was angry all the time and I used to hear a disturbing noise “chichichi” which looks like insects are moving inside my head*. *… Now*, *I suspect depression when clients are behaving inappropriately*, *when they say ‘I lost my card’ while holding the card on their hand (when they loss their attention)*, *when they frequently reported forgetting to take their medications*.”

#### Perceived severity of depression

Participants debated on ranking the severity of the condition presented in the case study. Half of the participants said that the condition was moderate.

*AS1*: *“I would say Selam’s illness is moderate*. *Because*, *depression comes with one’s own problem (fault)*. *Therefore*, *it should be solved by herself*. *If she explains her problem to others*, *she will become fine*. *She is keeping problems secret*. *She must start talking then she could improve*.”

*SU5*: *“Her illness is moderate*. *Why I said ‘her illness is moderate’*, *because she is not crazy like others on the street*. *What she actually did is just she sat down and think about herself*.”

The remaining half classified depression as a severe problem. The rationale for classifying the condition as a severe problem was the presence of hopelessness and suicidal ideation in the case study.

*SU6*: *“She (Mrs*. *Selam) becomes hopeless in life so that her illness is severe*. *Because hopelessness itself is enough to classify her illness as severe*. *She is not eating*, *and she is losing weight*. *When the hopelessness is added to these*, *it makes her illness so severe*.”

*AS3*: *“I would say her illness is severe*. *She has a wish to die*. *She would die if she were in the right time and place*. *Therefore*, *her illness is severe*.”

#### Perceived impact

All participants agreed that depression has several impacts at individual and family level, especially when it comes to PLWHA. Most of them reported that the immediate consequence of depression is ART non-adherence which leads to rapid increase in viral load count.

In relation to the impact of depression at individual level, a 35 years old female adherence supporter said, *AS2*: *“People with depression do not take their medications properly*. *At the time of depression*, *not only avoiding to take their medication on time but also they dislike to eat meals*. *At that miserable time of hopelessness*, *they would prefer to die rather than extending their life a bit longer with the support of medications*. *Therefore*, *it is quite obvious that people with depression discontinue their medication commonly*.*”*

Similarly, a 40 years old case manager added, *CM2*: *“… The main impact is on the medication adherence*. *Medications may not be taken properly*. *If medications are not taken properly*, *drug resistance follows*, *and their viral load increases rapidly*. *They forget what they are told to do so*. *…*.. *they may take medications twice when they were ordered to take once per day*. *…*. *They may skip taking the medications because they think that they already have taken their daily medications*. *There were people who came to the clinic within ten days after finishing all the medications prescribed for one month”*.

Moreover, many study participants described many long-term impacts of depression such as separation, family disintegration, non-functioning and poverty.

*SU6*: *“It is very difficult*. *HIV/AIDS is affecting our social life especially when it is combined with depression*. *We distance ourselves from our friends and families*. *I used to hate my family and myself*. *I guess I was depressed that time*.”

A similar idea was reflected from another adherence supporter as follows:

*AS1*: *“First it leads to self-stigmatization [self-isolation from social affairs] from the community*. *Second*, *it leads to economic problems because people with depression cannot work properly*. *Third*, *they become incapable to support their family*. *Afterwards*, *they may be separated from their family*.”

Substance use was reported to be one of the long-term impacts of depression.

*CM4*: *“People with depression get into a different life style*, *for example they may start using substances and drugs*. *They start cigarette smoking*, *khat chewing and alcohol drinking habits*”

Some other participants reported that the risk of committing suicide is much higher among depressed PLWHA. One of the participants narrated the risk of committing suicide as follows.

*AS3*: *“She (Mrs*. *Selam) has a wish to die so the impact of depression is clear*. *She could die if she is not treated on time*.”

#### Pathways to care

Many participants reported that Holy water/faith healers, witchcrafts and traditional healers are preferred pathways for treatment in the community. Some of the participants believe that bathing or drinking with ‘tsebel’ (Holy water)–which is a common traditional healing treatment in Ethiopia, could help to treat depression and other mental problems. Holy water is water blessed by a religious father, priest or member of the clergy.

*CM6*: *“When I was mentally sick*, *I went to Holy water ‘Tsebel bet’*, *and I visited traditional healers including witchcraft men several times*. *Because the community believe that mental problems that are acquired from ancestors or caused by evil attack or spiritual possession can be treated only by Holy water and traditional healers*. *To be honest*, *finally*, *I was treated by Holy water called ‘Abune Hara tsebel’*”.

CM6 continued… *“There is a saying that ‘a witchcraft man does not have any treatment for his illness*, *however the community still prefer to go to witchcraft to get help for mental problems*”

#### Treatment preference

When focus group participants were asked what possible interventions are needed for the condition presented in the case study, all of them spontaneously stated that counselling can be helpful to treat the described case (depression). All of the focus group participants emphasized the need to include psychological treatments with HIV care. Most of them had a basic understanding of psychological treatments and described these in different ways, such as counselling, psychological therapy, talk therapy, active listening and psychosocial support. All focus group participants agreed on the benefits of psychological treatments for depression for PLWHA.

*CM4*: *“What comes to my mind all the time is why psychologists were not included in the team when the HIV care was started*. *This was a must to include psychologists in the HIV care*. *I know how many people we lost by suicide*, *I know how many people became mentally sick and I know how many people discontinued their ART follow up*. *The past is passed once*. *But we should start working together for the future*.”

Active listening was reported as a tool used to engage depressed PLWHA in the process of counselling.

*AS3*: *“Mrs*. *Selam needs someone who can listen to her*. *Active listening treats many people who are in trouble and those suffering from excessive worry and sadness*.”

Another participant added a similar idea.

*CM5*: *“From my experience as a case manager*, *I came to understand that listening to their problem alone treats half of their condition*. *…*. *We come up with solutions together*, *such as creating a supporting system in every aspect–socially*, *psychologically and even economically*.”

Some of the participants narrated that counselling can be as effective as antidepressant medications.

*CM6*: *“I believe that counselling can treat depression as well as the medications*. *As to me*, *it is not right to prescribe medications when clients tell us stories such as anger*, *forgetfulness*, *and sadness*. *Medications should be prescribed if and only if counselling failed to treat depression*.”

This point was further elaborated by another participant:

*CM2*: *“Counselling should come first before prescribing antidepressants*. *I mean we should provide adequate counselling and see the outcome before giving medication*. *First*, *the client should believe in any treatment*. *Therefore*, *counselling can help a client to be aware and adhere with any type of treatment*.”

Many participants believe that peer counselling can help people with depression.

*SU3*: *“A psychosocial group should be established for depressed people as well*. *Peer-counselling can help to identify solutions for our day to day problems*. *We can learn a lot from others*.”

#### Acceptability of group IPT

Study participants were asked what format of counselling would be preferred by PLWHA and they supported group counselling as can help clients to make decisions and to learn social skills. Some of them described how much they had benefited from a group-based youth HIV counseling programs a few years back.

*AS1*: *“People with the same problem (HIV) gain energy when they are together*. *They could solve problems and make decisions together*. *There is a saying ‘Dire biaber anbessa yasir’ [a mass of thread can tie a lion] which means you can win any problem when you are together*. *There is nothing that can make clients afraid to talk when they are in group*. *Because all of us share the same problem and all have passed through difficult life circumstances*. *I believe they will become eager to talk about their life stories during the group sessions*.”

The above opinion was supported by a number of other participants.

*SU3*: *“I am sure people like the group counselling*. *It can be acceptable*. *For example*, *the psychosocial group helped me a lot when I was a child*. *I acquired HIV from my mother and I was not aware from whom I acquired it for so long*. *After I joined group of peers*, *I have learned a lot*. *I have got a lot of support from my peers related to HIV/AIDS*. *Everyone in the group was happy to support me at that difficult time*. *Moreover*, *such group counselling program should be started for adults too*.”

*SU4*: *“It is a great opportunity when people with the same problem talk to each other*. *People have a tradition of discussing in groups in this clinic*. *They exchange ideas with their friends*. *I believe the approach can be acceptable*.”

#### Delivering agent

Focus group participants were asked who would be preferred to facilitate group sessions. All study participants strongly proposed the use of trained peer counsellors to facilitate psychological interventions.

*AS1*: *“Peer counsellors can communicate with clients very easily*, *including with their gestures*.”

Focus group participants argued that the use of professionals suggests “severe mental health problem” and they were concerned about such labelling.

*CM2*: *“The group counselling you planned to introduce would be great if it is provided by trained peers*. *Peers can be selected from clients*. *…… Sometimes*, *when we take mentally disturbed clients to other professionals*, *their illness worsens*. *For example*, *if I am referred to a psychiatrist or psychologist*, *I will be worried more and more about the severity of my illness*. *I may say*, *‘why they refer me…am I getting crazy*?*’*. *… Depressed clients can be treated effectively if they are counselled by their peers*. *They can get a lot more lessons and problem-solving techniques from peers than from professionals*.”

One of the focus group participants emphasized on the need to get competent group facilitators to make the intervention effective.

*SU4*: *“There should be a competent person to facilitate group counselling among us*. *To make the group counselling effective*, *a better person should be selected to lead the group counselling*.”

#### Context

Most of the participants suggested that the group counselling should be conducted inside rooms not in open spaces.

*SU5*: *“People may not be comfortable when group sessions are conducted in an open space*. *Therefore*, *an ideal meeting space should be selected*. *The group sessions should be conducted inside a room that should not be accessible by other people who are not a member of the group*.”

Not everyone was happy on the idea to have separate groups by gender as they felt this was not an acceptable approach. As supported by the quotes below, all focus group participants recommended that men and women be mixed in one group.

AS2 (Female): *“As to me*, *it is not fair to make a separate group for males and females*. *Because all are matured and are family leaders and passed through difficult life circumstances*. *The experience of men and women complements each other and that would be great for the group harmony*.*”*

CM2 (Male): *“I do not completely agree on the idea to have separate groups by gender*. *I think the mixture of the two genders would be a great tool to facilitate team support*. *They can share ideas especially on issues related to marital problems*.*”*

However, most participants agreed the need to make separate groups by age. They believe that children (below 18 years old) and adults should be assigned in separate groups. Their rationale for having separate groups by age was because young children may not talk freely in front of adults.

CM3 (Female): *“…*.. *Clients can be categorized by age*, *such as below and above 18 years old*. *Eighteen-year-old man can think similarly to 60-year-old man*, *so they are the same*. *We should not make too many categories by age difference*.*”*

There was a debate on frequency of sessions and meeting days. Some participants suggested having group counselling sessions once per month and during the weekend days, either Saturday or Sunday. This idea was not accepted by the majority. Many of the participants argued that the learned skills and techniques of the therapy will be forgotten if the group sessions are conducted every month. They rather suggested to make group sessions at least once a week.

SU2 (Male): *“Having a group counselling once per week for 2 months is simple to attend for me*. *But what I would advise is the group sessions should be conducted in a place that should be convenient for many of the group members*. *The meeting place should be chosen with agreement of the group members*.*”*

The weekend days were not accepted by many of the participants. Their concern was related to confidentiality. They debated that coming to group sessions every weekend may elicit questions from people around them.

AS2 (Female): *“The weekend days may not be convenient for many of us*. *The group counselling should be integrated with regular ART care on the working days from Monday to Friday*.*”*

#### Potential barriers

Participants discussed potential barriers to implementing the group counselling for PLWHA. Lack of transportation access, denial of depression, high case load at the ART clinic, lack of trained staff, stigma and availability of convenient meeting spaces were identified as potential barriers to implement the group counselling.

*CM4*: *“We have clients who are coming from different rural areas far from here*. *I am afraid that they cannot pay fees for their transportation or they may not have easy access to transportation*.”

*SU6*: *“People may not accept their illness*. *They may say ‘I do not have depression’*. *This is a knowledge gap that needs a lot of work to change their attitude*. *First*, *they should believe they are sick*.”

## Discussion

This study investigated explanatory models of depression and treatment contextualization for depressed PLWHA in Northwest Ethiopia. It is clear that depression has a name given as ‘Dibirt’ [depression] or ‘Ye Dibirt chigir’ [depression problem] by most of the study participants. Some study participants reported that ‘Medebet’ [depression] is a name used by mental health professionals in Ethiopia to describe depression, but they believe that the general population may not understand the term ‘Medebet’. Study participants believed that depression has several impacts at individual and family levels for PLWHA, and that it is one of the factors leading to non-adherence to ART, increasing drug resistance, high viral load, disability, poverty and death. Given this understanding, participants strongly supported the use of psychological treatments for depression among PLWHA.

Important sub-themes emerged from the perceived causes, symptoms and treatment needs. We identified that depression is understood to be predominantly caused by psychosocial problems among PLWHA. Most identified psychosocial problems as the target areas (problem areas) for interpersonal therapy, including conflict, life change and loneliness [33]. Our study supports reports of previous studies [45, 46] that PLWHA attribute depressive symptoms to psychosocial problems. The lived experience of PLWHA confirms the inevitable presence of interpersonal stressors, such as conflict within a family, life changes in relation to acquisition of HIV/AIDS and stigma attached to HIV/AIDS, that contribute to the onset of depression [29, 46, 47]. The findings of our study confirmed the relevance of spiritual beliefs in relation to the onset of depression that is supported by results of previous studies [34, 35].

In our study, affective symptoms were the main ones with few mentions of somatic symptoms described whereas in previous studies somatic symptoms were predominantly reported by PLWHA [6, 19, 45]. Our study participants may have been prompted to focus on affective symptoms of depression after listening to the presented case study. Furthermore, the mhGAP training that some of the participants had received five years back may have helped them to understand depression as an affective disorder. However, most study participants described depressive symptoms from their lived experience.

Study participants believed that peer counselling can be helpful for people suffering from depression. They described their prior experience on a youth HIV counselling program which brought social support and social connection for many of them. They emphasized the benefits of group-based peer counselling that can help with socialization and social support. This finding is consistent with previous studies which have reported that group-based treatments have been preferred by depressed PLWHA in sub-Sahara Africa [11, 14, 29, 38] as they enhance social networking, social support and allow participants to learn new social skills [14]. Furthermore, they believe that group-based treatments provide feelings of empowerment, acceptance and companionship [29]. Study participants perceived the benefits of spiritual treatments such as Holy water for healing and recovery from mental conditions including depression. This indicates that spiritual interventions of any faith and religion could have important implications for treatment of depression for PLWHA in Ethiopia. Therefore, developing/adapting evidence-based treatments that target psychosocial needs and incorporating spiritual elements into treatment models may improve acceptability and efficacy of psychological treatments particularly for PLWHA. This finding is supported by results from several studies that incorporating spiritual elements with psychological interventions can help to treat severe psychological problems associated with life threatening health conditions [34–36, 48]. However, peer evaluation of counsellors, refresher training and regular supervision by health professionals should be in place.

Group IPT could be an acceptable treatment for depressed PLWHA in the ART settings but requires structural and content adaptation within the context of existing health care systems [2, 7]. The findings from our study suggest that a strong emphasis should be given to selection of delivering agents and identifying meeting spaces. Study participants from all focus group discussions emphasized that case managers and adherence supporters should facilitate group sessions. Case managers and adherence supporters were perceived as trusted peers and study participants believed that they can understand psychosocial problems of PLWHA. There was some concern that they may not have the competency to deliver the group IPT since most of them had secondary education only. However, several studies have shown successful reduction of depressive symptoms using lay counselors [38, 49–52].

The structure of groups was recommended to be age specific. Adults 18 years and above should not be mixed with adolescents. The reason was that adolescents may not feel free to talk about their personal stories in front of adults during the group sessions. However, all men and women study participants recommended the mixing of men and women clients in one group. They believed that mixing men and women can facilitate social support and group harmony. The participants felt that all, including clients and facilitators, should be PLWHA so that common feature would be sufficient to ensure cohesion for mixed groups. This finding is different from the previous study report by Nakimuli-Mpungu et al [14] that groups should be same gender because women may fear to talk their mind in front of men [14]. Cultural and educational status of group participants may affect the group atmosphere. For example, in a patriarchal and largely illiterate community, mixing men and women in one group may not be successful.

In relation to group size, six to ten clients per group was seen as acceptable by study participants, with the size of the venue determining the actual size. Large meeting spaces/rooms can accommodate up to ten individuals in one group. However, large group sizes require long session duration to allocate sufficient time for each client, otherwise, the therapy may not be delivered as intended. In terms of session frequency, one group session per week was welcomed by many of the study participants. Running group sessions every week allows sufficient time for clients to exercise the learned skills at home and it may help them to engage actively in each session. This finding is in agreement with the WHO group IPT guideline and with the previous study report from Uganda [12, 14]. With the above conditions, the group IPT could become an acceptable treatment for depression for PLWHA in Ethiopia.

In relation to confidentiality, study participants preferred indoor settings for group meetings as they prevent intrusion from non-members. In addition, as described above, the timing is important to ensure confidentiality, that is not on weekends. Hence, future studies should consider confidentiality issues, such as venue and timing of group sessions, when implementing psychological treatments for PLWHA. Study participants described potential barriers to conducting group IPT in the ART settings. For example, lack of trained facilitators was described as a barrier to conduct the intervention by all/many of the groups. The other anticipated barrier was lack of access to transportation and travel fatigue for group members. This is a practical barrier common to many interventions in low income countries [53–55]. Arranging meeting spaces in the nearest setting may be an option. However, clients may not prefer the nearest settings to their home due to fear of stigma and discrimination in the community. But lack of transport does mean that interventions will remain inaccessible for the majority of poor and disadvantaged communities.

Finally, we found that understanding local explanatory models of depression could maximize acceptability and effectiveness of group IPT for treatment of depression for PLWHA in Northwest Ethiopia. Thus, local terminologies used to describe depression such as “Dibirt” [depression] or “Y’Dibirt chigir” [depression problem] should be incorporated into the group IPT manual and other intervention guidelines. In addition, group IPT facilitators should be trained to understand local explanatory models, including the biological, psychological, social and spiritual explanations that local people have for depression.

### Limitations

This qualitative study has several limitations that should be noted. Firstly, we used a relatively small number of participants with only one focus group per stakeholder category. Secondly, the study participants may have been biased by the case vignette presented during the focus group discussion. Although there was strong agreement on most of the themes, the results found from *perceived symptoms of depression* may be influenced by the story of the case vignette. Third, our study used focus group discussions for data collection, therefore it may have limited exploration of sensitive issues such as sexual abuse and harassment.

## Conclusion

This study highlighted that a number of psychosocial problems were perceived as contributing to the onset of depression among PLWHA in Northwest Ethiopia. PLWHA believe that depression affects adherence to ART and leads to increasing drug resistance, high viral load, disability, poverty and death. Group-based psychological treatments delivered by trained peers were the preferred intervention approaches for depressed PLWHA. Group IPT was seen as acceptable for PLWHA. However, special attention should be given to the adaptation and contextualization of the intervention particularly on incorporation of local explanatory models, selection of facilitators, selection of meeting spaces and group structure. We recommend further studies to investigate whether to include spiritual elements with core principles of group IPT especially for PLWHA in Ethiopia. The current study findings will be used to assess feasibility and acceptability trials of group IPT in large HIV populations in Ethiopia.

## Supporting information

S1 File A summary of interview guide in the Amharic language. (DOCX)

S2 File A summary of interview guide in the English language. (DOCX)

S3 File Case vignette in Amharic. (DOCX)

S4 File Case vignette in English. (DOCX)
