# Supplementary material for: Adaptation of the WHO group interpersonal therapy for people living with HIV/AIDS in Northwest Ethiopia: A qualitative study
Source: PLoS One. 2020 Aug 27;15(8):e0238321. doi: 10.1371/journal.pone.0238321 (PMC7451549; doi:10.1371/journal.pone.0238321)
Supplement: S1 File — (DOCX) [file pone.0238321.s001.docx]

**አማርኛ ቃለ-መጠይቅ**

**በፈለገ-ህይዎት ሪፈራል ሆስፒታል ውስጥ የኤችአይቪ/ኤድስ ህክመና ተከታታዮች እና የአቻ ለአቻ አማካሪዎች ጋር የሚደረግ የቃለ-መጠይቅ መግቢያ**

**የቃለ-መጠይቁ አላማ፡** የመጀመሪው የመጠይቁ ክፍል አላማ በፈለገ-ህይዎት ሪፈራል ሆስፒታል ውስጥ የኤችአይቪ/ኤድስ ህክምና ክትትል ላይ ያሉ እና የአቻ ለአቻ አማካሪዎች በድብረት ህመም ምልክቶች ላይ ያላቸውን ግንዛቤ እና አረዳድ ለማዎቅ የሚደረግ መጠይቅ ነው፡፡ ሁለተኛው ክፍል ደግሞ የድብርት ምልክቶች ያሉባቸው የኤችአይቪ ህክምና ተከታታዮች ምን አይነት የህክምና እርዳታ እንደሚፈልጉ ለማዎቅና ህክምናውን በፈለገ-ህይዎት ሆስፒታል ውስጥ ለማመቻቸት የሚደረግ መጠይቅ ነው፡፡

**መመሪያ**

**እንኳን ድህና መጣችሁ እና መግቢያ**

ተሳታፊዎች በዚህ መጠይቅ ለመሳተፍ ስለመጡ ማመስገን

**ፕረጀክቱን ማብራራት**፡ እዚህ የተገኘነው ከእናነተ ጋር ለመስራት እና የድብርት ምልክቶችን ለማከም የቡድን የምክክር አገልግሎትን በዚህ ክሊኒክ ውስጥ ለመጀመር አስበን ነው፡፡ ስለሆነም ይህን ፕሮጀክት ውጤታማ ለማድረግ እና አገልግሎቱን ለማስጀመር የእናነተ ድጋፍ በጣም አስፈላጊ ነው፡፡

**ክፍል ሀ፡ በፈለገ ህይዎት ሆስፒታል የኤችአይቪ ህክምና ተከታታዮች እና የአቻ ለአቻ አማካሪዎች በድብርት ህመም ምልክቶች ላይ ያላቸውን ግንዛቤ ለመዳሰስ**

በመጀመሪ ሰላም የምትባል ሴት የገጠማትን ችግር እናያለን፡፡ ከዚያም የሰላምን ችግሮች መሰረት በማድረግ በሚነሱ የመዎያያ ሃሳቦች ላይ ዘርዘር ያለ ውይይት እናደርጋለን፡፡

1. ሰላም ምን ምን ችግሮች ገጠሟት? (ምልክቶቹን እንዴት እንደተረዷቸውና ከምን ጋር እንደሚያያዙ መጠየቅ፤ ለምሳሌ፡ ከመተት፤ ውለሽ ወይም ከውጥረት ጋር በተያያዘ)
2. እንደሰላም አይነት ችግሮችን ማህበረሰቡ ምን ብሎ ይገልፃቸዋል/ይጠራቸዋል? (ሰላም ላይ ከታዩት ምልክቶ በተጨማሪ ምን ምን አይነት የድብርት ምልክቶችን ያውቃሉ? እነዚህ ምልክቶች በሰላም እና በሌሎች ችግሩ ባለባቸው ሰዎች ህይዎት ላይ ምና አይነት ተፅዕኖ ያመጣሉ? አነዚህ ምልክቶች ለምን ያክል ግዜ ሊቆዩ ይችላሉ?)
3. የነዚህ ምልክቶች አምጭ ምክንያቶች ምነድን ናቸው ብለው ያስባሉ? (ለምሳሌ፡ ምን አይነት ሁኔታዎች/ችግሮች ናቸው የድብርት ምልክቶችን የሚያስነሱት? የሰወች እምነትና አመለካከት ሊሆን ይችላል? ለምሳሌ ለራስ ያለ ዝቅተኛ አመለካከት)
4. እንዲህ አይነት የድብርት ምልክቶች በኤችአይቪ ህሙማን ላይ የተለመዱ ናቸው ብለው ያስባሉ? የኤችአይቪ ህከምና ተከታታዮች ከሌሎች ሰዎች በበለጠ ለእንደነዚህ አይነት የድብርት ምልክቶች ተጋላጭ የሆኑበት ምክንያቶች ምንድን ናቸው? ለምን? እስኪ ሰፋ አድርገን እንውያበት::
5. ሰዎች እንዲህ አይነት ችግር ሲገጥማቸው እርዳታ ፍለጋ የት ይሄዳሉ?
6. የድብርት ምልክቶች የኤችአይቪ ህክምና ላይ ተፅዕኖ ያመጣሉ ብለው ያስባሉ? እንዴት? እስኪ ያብራሩት::

**ወደ ሚቀጥለው ክፍል ከመሄዳችን በፊት መጨመር የሚፈልጉት ሀሳብ ይኖር ይሆን?**

**ክፍል ለ፡ ሊተገበሩ የሚችሉ የህክምና አገልግሎቶችን በተመለከተ የሚደረግ የዳሰሳ ጥናት**

አሁን ደግሞ የድብርት ምልክቶች ያሉባቸው የኤችአይቪ ህክምና ተከታታዮችን ለመርዳት ስለሚደረጉ የህክምና አገልግሎቶች እንነጋገር፤

1. በፈለገ-ህይዎት ሆስፒታል ውስጥ የድብርት ምልክቶች ያሉባቸው የኤችአይቪ ህክምና ተከታታዮችን ለመርዳት ምን አይነት የህክምና አገልግሎት ተመራጭ ነው? ለምን?
2. የድብርት ምልክቶች ያሉባቸው የኤችአይቪ ህክምና ተከታታዮች ምን አይነት ተጨማሪ ድጋፍ የሚፈልጉ ይመስልዎታል?
3. ስለ ስነ-ልቦና የምክር ህክምና ምን ያውቃሉ? ምነ ምን አይነት የስነ-ልቦና ህክምናዎችን ያውቃሉ?

አሁን ደግሞ ስለ ቡድን የምክክር ህክምና ልንገራችሁ:: የቡድን የምክክር ህክምና የድብርት ምልክቶችች ከሚያክሙ የስነ-ልቦና ህክምናዎች ውስጥ አንዱ ነው:: ይህ ህክምና ከሚከተሉት አራት ምክንያቶች በአንዱ ወይም ከዚያ በላይ ምክንያቶች የመጡ የድብርት ምልክቶችን ለማከም ይጠቅማል:: እነሱም፡- 1) የቅርብ ወዳጃቸውን/ዘመዳቸውን በሞት ለተነጠቁና በጥልቅ ሀዘን ምክንያት የድብርት ምልክቶች ለያዛቸው ሰዎች 2) በጭቅጨቅና አለመስማማት ምክንያት 3) በህይዎት ዉጣውረድ ምክንያት ለምሳሌ ከስራ መባረር፤ ፍች መፈፀም፡ መለያየት ወይም ስደት 4) በብቸኝነት/ከማህበራዊ ህይዎት መገለልን የሚመለከቱ ናቸው፡፡ በዚህ የቡድን የምክክር ህክምና ግዜ ታካሚዎች በቡድን ሆነው ስለገጠማቸው ማህበራዊ እና ሌሎችም ችግሮች ይወያያሉ፡፡ የመፍትሄ አቅጠጫ ያበጃሉ፤ ለመፍትሄውም አብረው በጋራ ይሰራሉ፤ ከባለሙያም ድጋፍ እና አቅጣጫ ይሰጣቸዋል::

ስለዚህ ከላይ ባየነው የህክምና አገልግሎት (የቡድን የምክክር ህክምና) መሰረት የሚከተሉትን ሀሳቦች እንዎያይባቸው፤

1. ስለ የምክክር ህክምና ካሁን በፊት ሰምተው ያውቃሉ? ስለሆነም ይህን የቡድን የምክክር ህክምና በዚህ ሆስፒታል ውስጥ የድብረት ምልክቶች ላሉባቸው የኤችአያቪ ህክምና ተከታታዮችን ለማከም ብንጠቀመው ምን ይመስልዎታል?

- ተቀባይነቱን በተመለከተ ምን ያስባሉ? ተገቢነቱን እስኪ አብራርተው ይንገሩኝ፡፡
- ተግባራዊ ማድረግ ይቻል ይሆን? የማይሆን ከሆነስ ለምን? ተግባራዊ ለማድረግ ምን መደረግ አለበት ይላሉ?

ምን አይነት ህክምና ቢሰጥ የድብርት ምልክቶችን ማከም እንደሚቻል ነግረውኛል:: አሁን ደግሞ ህክምናው እንዴት፤ በማን፤ መቼ መቼ፤ ለምን ያክል ግዜ እና የት ቦታ ቢሰጥ ይሻላል በሚሉ ሃሳቦች ዙሪያ እንነጋገር፤

የመዎያያ ሃሳቦች፡-

- በሳምንት ስንት ግዜና በስንት ሰዓት አካባቢ፤
- የመገናኛ ቦታው የት ቢሆን ይመርጣሉ?
- የአንድ ግዜ ስብሰባ ለምን ያክል ግዜ (ሰዓት)፡ ለምን ያክል ክፍለ-ግዜ ይሰጥ?
- የህክመና ቡድኑ ሲዋቀር ፆታን፤ ዕድሜን፤ ሙያን፤ አካባቢን ወይስ ምንን መሰርት ቢያደርግ ጥሩ ነው ብለው ያስባሉ?
- ህክምናውን ማን ቢሰጥ/ቢያስተባብር ይሻላል (በፆታ፤ በዕድሜ፤ በትምህርት ደረጃ)?
- በቡድን ወይስ በግለሰብ ደረጃ?

እስኪ እንዲህ አይነት ህክምና በሚጀመርበት ወቅት ምን ምን አይነት ችግሮች ሊገጥሙ ይቸላሉ? በታካሚዎች ተቀባይነት እንዲኖረውና አመች እንዲሆን ምን ቢደረግ ይሻላል ይላሉ?

**ውይይታችን ከመጨረሳችን በፊት መጨመር የሚፈልጉት ሀሳብ ይኖር ይሆን?**

**በጣም አመሰግናለሁ!!**
