# Supplementary material for: Adaptation of the WHO group interpersonal therapy for people living with HIV/AIDS in Northwest Ethiopia: A qualitative study
Source: PLoS One. 2020 Aug 27;15(8):e0238321. doi: 10.1371/journal.pone.0238321 (PMC7451549; doi:10.1371/journal.pone.0238321)
Supplement: S2 File — (DOCX) [file pone.0238321.s002.docx]

**Interview guide for interviews of People living with HIV/AIDS (PWHA) and adherence counselors**

**Objective of questionnaire: Section one aimed to explore conceptualization of depressive symptoms by People Living with HIV/AIDS (PWHA) and adherence counselors who are attending their clinical follow up in the ART clinic in Felegehiwot Hospital. Section B aimed to identify preferred treatment strategy to help depressed PWHA in Felegehiwot referral hospital.**

**Guidelines**

Welcome and introduction

Thank participants for agreeing to participate

Explaining the project: We are wanting to work with you at the clinic to find out ways to start Group IPT counseling to depressed people with HIV/AIDS.

Explaining the rationale for the interview: We are doing this project so that we can find ways to start the provision of mental health services (counselling) to many more people.

**Section A. Depression Explanatory Model (DEM) for PWHA**

1. **Let’s listen a case presentation about stories of Selam then we will discuss about her story later (the case will be displayed on a projector). After listening the stories of Selam (“*Now, let’s discuss how you understood Selam’s story*”)**
2. Can you explain to me what Selam’s problems are? (*Probe* for understanding e.g., is it understood as witchcraft, stress etc, if it is any of these things, how do they work eg how does the witchcraft work, how does the stress work?, etc ).
3. What are the different words that you and other people use to describe Selam’s problem? *Probing* (What other symptoms do people have who are depressed and how do these symptoms affect her life? How long do these symptoms usually last?)
4. What do you think causes these feelings? (For example, is it specific events or situations that trigger or start the symptoms, or is it people’s beliefs, or other things)
5. How common are depressive symptoms in PWHA? Are PWHA more likely to be depressed than other people? If so, why?
6. Where do they seek help?

**Section B. Available intervention strategies (for adherence counselors)**

1. **Let’s talk now about providing services to help depressed PWHA in Felegehiwot hospital.**
2. What are the best treatment options available to help depressed PWHA in Felegehiwot hospital?
3. What additional support do you think they need?
4. What do you know about psychological treatments?

Now let me tell you about Group Interpersonal Therapy (Group IPT). Group IPT is one of psychological treatments used to treat depressive symptoms. It focuses to alleviate depressive symptoms triggered by one or more of these four problems: 1) grief/loss of beloved one’s, 2) Dispute/disagreement, 3) Life change including divorce, separation, migration, job loss, and 4) loneliness/separation. Depressed people will meet in group to share their personal problems that are causing their depressive symptoms, to share coping skills and to set goals to solve difficult situations. Therefore, depressive symptoms can be treated when those triggering factors solved using Group IPT.

Based on the above description of Group IPT, let’s discuss the following ideas.

1. Do you know about it before and if yes what are your thoughts about expanding it for larger population Ethiopia?

- Think of its acceptability and appropriateness.
- Is it feasible in this context and if not, how can we make it feasible?

You’ve told me about what types of treatments you think may work for PWHA with depressive symptoms. Let’s talk about how the treatment should be provided. For example, who should provide it, where it should take place, how often, and so on.

Probes:

- What time of day and week
- Where?
- How long the sessions should be and how many sessions
- Who should conduct the therapy as counselor? (gender, age, qualification, locality, relationship/ community relations).
- Group or individual

Let’s think about any problems in running such therapy sessions.

Probes:

- What would make it acceptable to people
- Would it be convenient for people?
- How can any problems be overcome?

**I thank you so much!!!**
