# Supplementary material for: Adaptation of the WHO group interpersonal therapy for people living with HIV/AIDS in Northwest Ethiopia: A qualitative study
Source: PLoS One. 2020 Aug 27;15(8):e0238321. doi: 10.1371/journal.pone.0238321 (PMC7451549; doi:10.1371/journal.pone.0238321)
Supplement: S3 File — (DOCX) [file pone.0238321.s003.docx]

**የሰላም ግለ-ታሪክ**

ሰላም በባህር ዳር ከተማ የምትኖር የ30 አመት የቤት እመቤት ናት፡፡ ባለፉት ጥቂት ሳምንታት ባልተለመደ ሁኔታ ከፍተኛ ሀዘን እና ስቃይ እየተሰማት ነው፡፡ ምንም ነገር አያስደስታትም፤ ሁሉም ነገር አስጠልቷታል፡፡ ሁል ግዜ ከፍተኛ ድካም ቢሰማትም እንቅልፍ ግን አይዛትም፡፡ ሰላም ምግብ መውሰድ ካቆመች ብዙ ግዜ ሆናት፤ ስለሆነም ክብደቷ በጣም ቀንሷል፡፡ ሃሳቧን ሰብስባ የቤት ስራዋን መከወን ተሰኗታል፤ ቀላል ውሳኔዎችን ለመወሰን እንኳን ተቸግራለች፡፡ በፊት በጣም ቀላል የነበሩት የቤት ለቤት ስራዎች ለምሳሌ:- እንጀራ መጋገር፤ ወጥ መስራት፤ ቤት ማፅዳት፤እቃና ልብስ ማጠብ እነኳን እንደ ተራራ ከብደዋታል፡፡ አንድ ቀን በተከፋ ስሜት ውስጥ ሆና ለባለቤቷ እንዲህ አለችው “ብቸኝንት ይሰማኘኛል፤ በሁሉም ነገር ተስፋ ቆርጫለሁ፤ የሆነ ነገር መጥቶ ቢገለኝ ምን አለ::” የዚህን ግዜ ነው ባለቤቷ የሰላም የጤንነት ሁኔታ አሳሳቢ ደረጃ ላይ መድረሱን የተረዳው::
