# Supplementary material for: Adaptation of the WHO group interpersonal therapy for people living with HIV/AIDS in Northwest Ethiopia: A qualitative study
Source: PLoS One. 2020 Aug 27;15(8):e0238321. doi: 10.1371/journal.pone.0238321 (PMC7451549; doi:10.1371/journal.pone.0238321)
Supplement: S4 File — (DOCX) [file pone.0238321.s004.docx]

***Case vignette***

*Selam is 30 years old housewife. She has been feeling unusually sad and miserable for the last few weeks. She has lost interest in everything and she is not getting pleasure at all. Even though she feels tired all the time, she has trouble sleeping nearly every night. Selam does not feel like eating and has lost weight. She cannot keep her mind on housework and puts off making decisions. Even day-to-day tasks seem too much for her such as cooking, baking, house cleaning and other activities. Once she told her husband that she is feeling helpless, hopeless, guilty with unknown reasons so that she prefers to die in whatever means. This has come to the attention of her husband who is concerned about Selam’s death wish.*
